# Recent advances in prenatal genetic screening and testing

**DOI:** 10.12688/f1000research.9215.1

**Published:** 2016-10-28

**Authors:** Ignatia B. Van den Veyver

**Affiliations:** 1 Department of Obstetrics and Gynecology and Department of Molecular and Human Genetics, Baylor College of Medicine, Houston, Texas, 77030, USA

**Keywords:** prenatal screening, genetic abnormalities, pregnancy, risk

## Abstract

The introduction of new technologies has dramatically changed the current practice of prenatal screening and testing for genetic abnormalities in the fetus. Expanded carrier screening panels and non-invasive cell-free fetal DNA-based screening for aneuploidy and single-gene disorders, and more recently for subchromosomal abnormalities, have been introduced into prenatal care. More recently introduced technologies such as chromosomal microarray analysis and whole-exome sequencing can diagnose more genetic conditions on samples obtained through amniocentesis or chorionic villus sampling, including many disorders that cannot be screened for non-invasively. All of these options have benefits and limitations, and genetic counseling has become increasingly complex for providers who are responsible for guiding patients in their decisions about screening and testing before and during pregnancy.

## Introduction

For more than 30 years, identifying women at increased risk for pregnancies with Down syndrome has been the focus of prenatal screening programs that combine maternal age, levels of specific analytes in maternal serum, and ultrasound findings in the first or second trimester to derive a risk estimate for Down syndrome and secondarily for trisomy 18. These programs now reach a detection rate of up to 88–96% for Down syndrome and up to 85–95% for trisomy 18 ^1, 2^, depending on whether screening is performed in the first or second trimester of pregnancy, or both. In parallel, programs for universal parental carrier screening for autosomal recessive disorders, such as cystic fibrosis, as well as ethnicity-based carrier screening, such as for conditions more prevalent in the Ashkenazi Jewish population, were developed to identify parents at 25% risk of having an affected child with these disorders ^3^. Identified carrier couples can then choose preimplantation genetic diagnosis to avoid affected pregnancies, or prenatal diagnosis, allowing them to consider termination of affected pregnancies or be prepared for the birth of an affected child.

With recent technological advances in methods to identify numerical and structural chromosome abnormalities and point mutations, such as array-based copy-number analysis, also known as chromosomal microarray analysis (CMA), and next-generation sequencing (NGS), the screening for and diagnosis of genetic abnormalities in the fetus is undergoing an unprecedented rapid evolution ^4– 13^. In parallel, CMA and NGS have also accelerated the discovery of causes of intellectual disability, birth defects, and many rare genetic and genomic disorders ^14– 17^. This has motivated the development of expansive carrier screens for hundreds of genetic disorders at once as well as the development of non-invasive cell-free fetal DNA (cffDNA)-based screens for fetal chromosomal aneuploidy, subchromosomal abnormalities, and single-gene disorders. The availability of CMA- and NGS-based methods, such as targeted gene-panel sequencing and, recently, whole-exome sequencing (WES), has also resulted in the ability to diagnose more fetal genetic conditions from samples obtained through amniocentesis or chorionic villus sampling (CVS).

All of these new tests have created new, exciting opportunities for comprehensive prenatal diagnosis and screening, but they are accompanied by important challenges. Healthcare providers must consider the consequences of their rapid introduction into the clinic because of the still-limited knowledge about the test performance of some assays in routine clinical practice, concerns related to cost-conscious implementation of optimized screening and testing strategies, equal access, and appropriate selection of who will benefit most. The ever-increasing amount of genetic information that can be obtained preconceptionally and prenatally also brings about ethical and genetic counseling challenges ^9, 18– 21^.

## The introduction of chromosomal microarray analysis into prenatal diagnosis

The early goal of prenatal genetic screening was to identify women at increased risk for having a pregnancy with Down syndrome, resulting from an extra chromosome 21 (trisomy 21), the most common aneuploidy in liveborns, and, secondarily, Edwards syndrome (trisomy 18) and Patau syndrome (trisomy 13), so that a diagnostic amniocentesis (withdrawing amniotic fluid from inside the uterus that contains fetal cells) or CVS (obtaining a small sample from the placenta) can be offered to those at increased risk. For many years, the standard test on cultured cells from prenatally obtained amniotic fluid or CVS has been a karyotype (chromosome analysis) that can detect chromosomal aneuploidy (extra or missing chromosomes) and structural abnormalities larger than 5–10 megabases (Mb) in size. This is sometimes supplemented by fluorescence *in situ* hybridization (FISH) to rapidly test for a few common aneuploidies if an expedited diagnosis is desired. FISH with locus-specific probes was also the method of choice to test for smaller structural chromosomal abnormalities but requires knowledge about which locus might be of interest, and only a few loci can be investigated in a single assay.

This dramatically changed when CMA became available, in which fluorescently labeled DNA is hybridized to a slide that carries thousands of probes spread across the genome. Higher or lower fluorescence intensity coming from DNA hybridized to specific probes identifies regions that have extra or missing copies of DNA, respectively. CMA has a much higher resolution than karyotyping, spanning from entire chromosomes (aneuploidy), to deletions and duplications of just several kilobases (kb) or even single exons. It also does not require cell culture, thus results can be available faster. CMA is now the first-tier genetic diagnostic test for children and adults with multiple congenital anomalies, genetic syndromes, and intellectual and developmental disabilities, where its diagnostic yield is 15 to 20% ^22^. Widespread use of CMA for prenatal diagnosis lagged behind until results from a landmark multicenter trial sponsored by the National Institutes of Health, confirmed by other studies, demonstrated that CMA detects a clinically significant and potentially clinically significant copy number change in 1.7% of pregnancies with a normal karyotype and no observable fetal abnormalities; others have found a rate of 1% for clinically significant copy number variations (CNVs) ^23^. However, CMA also detects CNVs of uncertain clinical significance and that predispose to later-onset disorders in about 1% of cases (up to approximately 2%, depending on the study). This increases to 6% when there are congenital anomalies in the fetus ^6, 23^. CMA also performs better than a karyotype for the analysis of stillbirth samples ^24^. The American College of Obstetrics and Gynecology now recommends that CMA is offered as the first-line test when fetal abnormalities are present and for stillbirth samples ^25^. CMA is also better than karyotyping for genetic studies of early miscarriages. Although about 50% of miscarriages are aneuploid, some have subchromosomal abnormalities and standard karyotyping is compromised in 40% owing to culture failure or maternal-cell contamination ^26^.

The significantly higher detection rate of chromosomal abnormalities with CMA, along with recommendations that amniocentesis should be made available to all women ^27^, led to predictions that more women would accept the small risk of amniocentesis or CVS for this benefit, which in a recent meta-analysis was found to be 0.11% or 1:909 and 0.22% or 1:454, respectively ^28^, and not elevated compared to background in another recent study ^29^. However, new developments in cffDNA-based non-invasive screening of maternal plasma or fetal aneuploidy reversed this expected trend, with a dramatic decrease in the number of diagnostic procedures performed ^30^. Contributing to this decrease are a combination of assertive marketing of the new cffDNA-based tests by industry, incomplete understanding about the clinical performance and screening nature of cffDNA analysis, and a desire by women to avoid any potential risk to their pregnancies.

## How cell-free fetal DNA analysis has changed the approach to prenatal diagnosis of genetic and chromosomal abnormalities

An ideal prenatal genetic diagnostic test would be both non-invasive and comprehensive, capable of simultaneously detecting chromosomal aneuploidy, structural chromosomal abnormalities, and single-gene mutations. Early efforts in the 1990’s focused on isolating fetal cells and analyzing them for chromosomal aneuploidy, but the success rate was no better than standard maternal serum screening ^31^. This was primarily because these circulating fetal cells are rare and difficult to purify and the diagnostic tools, mostly single-cell FISH, were limited. When Lo *et al.* discovered in 1997 that male fetal DNA could be amplified by PCR from maternal plasma ^32^, attention shifted to the analysis of cffDNA from maternal plasma. Initially, PCR-based assays to identify fetal gender ^33^, fetal Rhesus genotype ^34– 39^, and mutations that cause paternally inherited or *de novo* single-gene disorders were developed, which is an ongoing field of active investigation ^40– 44^. In 2008, two groups reported that shotgun NGS of cell-free DNA (cfDNA) from maternal plasma, of which about 10% originates from the placenta and represents the fetal genome, can be used to determine if there is fetal aneuploidy by counting sequence tags mapped to each chromosome ^45, 46^. Following this, a number of technical and clinical validation studies collectively showed high sensitivity and specificity for the detection of Down syndrome and other common aneuploidies in pregnant women at increased risk for fetal aneuploidy ^47– 56^. Different technologies, one based on massively multiplexed PCR and another based on selection and sequencing of specific tags from chromosomes of interest, have also been developed and have similar performance ^57– 65^. Overall, cffDNA-based tests have a detection rate and false positive rate of 99.4% and 0.16%, respectively, for Down syndrome, 96.6% and 0.05% for trisomy 18, 86.4% and 0.09% for trisomy 13, and 89.5% and 0.20% for monosomy X ^1^. Those numbers were mostly obtained from studies in a high-risk population ^66^, where the positive predictive value (PPV) for common aneuploidies, such as trisomy 21, is high and does not take into account the small numbers of samples where no result was obtained. As a reminder, PPV indicates how often a positive test result reflects a true positive and depends on the prevalence of the condition in the population studied. The PPVs of cffDNA screening are lower in low-risk or average-risk populations ^52, 56, 59, 62, 65, 67– 69^ but still significantly better than those of the standard multiple marker serum screening algorithms ^1^. Together with reports from cytogenetic laboratories of relatively low confirmation rates in fetal samples studied because of positive cffDNA screening results ^70, 71^, this has raised concern that non-invasive cffDNA testing for aneuploidy is less accurate when applied in clinical practice than was expected based on the published validation studies, which is an issue that has not yet been completely resolved and is to some degree also platform dependent. This underscores the need for objective genetic counseling with emphasis on the screening nature and limitations on the accuracy of these tests. Some companies also offer cffDNA screening for twin pregnancies, but test performance is lower, in part because the cffDNA is a mixture of DNA from two fetuses ^72^. One study has shown that this also influences results when a twin pregnancy has very early loss of one fetus or “vanishing twin” and that cffDNA from the trophoblast of the demised twin can be found up to 8 weeks after the demise ^73^.

As experience with cffDNA screening grows, other unknowns and caveats have emerged that complicate pre- and post-test genetic counseling. Because circulating cffDNA derives from the trophoblast, confined placental mosaicism for a tested chromosomal abnormality, known from CVS studies to be present in about 1% ^74^, may result in a positive cffDNA test, but the fetus is unaffected ^72, 75– 77^ when follow-up diagnostic testing on amniotic fluid samples (preferred over CVS in these situations) is performed. Since cffDNA is admixed with a large excess of maternal cfDNA fragments, maternal mosaicism for the detected chromosomal abnormality in the mother ^72^ may also cause a positive cffDNA screening result. For example, low-level germline or acquired mosaicism for monosomy X has been well described ^78, 79^. Depending on which platform is used, <1 to 5% of the tests may fail, which has also been found to be associated with a higher risk for fetal aneuploidy ^80^. One cause of this could be low fetal fraction (i.e. the proportion of all the cfDNA in maternal plasma that is fetal) owing to placental abnormalities in some aneuploidies ^72, 81^. However, other more common causes for low fetal fraction are a high maternal body mass index or early gestational age ^72, 82, 83^, the reason that cffDNA screening is not recommended before 10 weeks’ gestation. Bianchi *et al.* first reported that rarely false positive cffDNA-screening results, particularly those suggestive of multiple aneuploidies or aneuploidy incompatible with embryonic or fetal development, may be associated with maternal malignancy, with the chromosomally abnormal cfDNA originating from tumor cells ^84– 86^. While this is of potential high clinical impact, it is not currently established what the optimal follow-up for such women should be. Other maternal reasons for abnormal cffDNA-screening results can be the presence of fibroids ^87^ or, in rare cases, transplanted organs.

After initial demonstration that microdeletions can be detected in cffDNA ^88– 92^, some providers now offer the option to add screening for selected clinically significant microdeletions and also rarer aneuploidies (trisomy 9, 16, and 22) ^93– 99^. One provider in the United States recently began offering genome-wide cffDNA screening for deletions and duplications of >7 Mb ^100, 101^. Rigorous clinical validation of these expanded cffDNA tests is problematic ^102, 103^ because these additional genetic conditions are each very rare and there is significant concern for high cumulative false positive and false negative rates.

To date, guidance offered by professional societies on cffDNA analysis state that they are screening tests and do not replace diagnostic testing ^1, 2, 104– 108^. Most, but not all ^105, 108^, also recommend offering it only to women at increased risk for aneuploidy, but all state that cffDNA screening for microdeletions has not yet been sufficiently clinically validated. Despite this, and likely because of intense marketing, many women are being offered cffDNA screening, irrespective of *a priori* risk, and the number of diagnostic procedures performed has dramatically declined. Many have voiced concern that this will result in failure to detect significant chromosomal abnormalities currently only detectable by karyotyping and CMA. In addition, when diagnostic testing is performed after positive standard first trimester combined screening, 17–30% of chromosomal abnormalities identified in the fetus are not those for which the screen was positive ^109, 110^ and would not be detectable by currently offered cffDNA screening tests. Although it has recently been argued that this is less frequently a concern ^111^, data from a study in which common aneuploidy-specific qfPCR as follow-up testing on amniotic fluid samples for an abnormal serum screening result was compared to karyotype analysis ^112^, and another retrospective analysis also indicated that other chromosomal abnormalities that would be missed by cfDNA screening can be responsible for abnormal maternal serum screening results ^113^, although at reported variable frequencies. Finally, reports that are not easy to confirm are also emerging that women have foregone confirmatory testing and made reproductive decisions based on cffDNA screening results alone ^114, 115^.

## The emergence of expanded carrier screening

Another recent development is in the area of carrier screening. For autosomal recessive genetic conditions to manifest, both copies (alleles) of a disease gene have to carry a deleterious mutation and carriers with only one mutant copy are unaffected. However, carriers for a deleterious mutation in the same gene have a 25% (1/4) risk with each pregnancy to have an affected child. Professional societies recommend reproductive carrier screening for a limited number of conditions, some of which pan-ethnically (e.g. spinal muscular atrophy) and some based on ethnicity (e.g. thalassemia, sickle cell disease, and conditions prevalent in the Ashkenazi Jewish population) ^116– 120^. These recommendations are based on consensus among experts that take into account disease severity, age of onset and prevalence, cost effectiveness, and the availability of therapies or other management options for affected individuals (including preimplantation or prenatal genetic diagnosis). Important limitations of this strategy for reproductive carrier screening include that many individuals do not have accurate knowledge of their ancestry, the increasing admixture in populations, and the focus of screening on more prevalent disorders, while other rarer but potentially equally or more severe conditions are not included ^121^.

To overcome such limitations, newer high-throughput mutation screening or sequencing methods have been developed that combine testing of multiple known disease genes in single “expanded” carrier tests and are beginning to be offered to women and their partners, irrespective of their ethnic background. Different companies are now offering such pan-ethnic expanded carrier screening panels, but there is variation in the number and identity of disorders screened for between different panels. Some also include copy number analysis for specific conditions and carrier screening of women for X-linked disorders with 50% risk of transmission to affected sons or to carrier daughters. These expanded carrier tests are a significant improvement compared to the smaller panels, but current cost and reimbursement policies limit universal access. In addition, as the number of genes included on these panels increases, 25% ^122^ or more ^123, 124^ of those screened will be identified as carriers, yet the chance that both reproductive partners carry mutations in the same gene remains low. The need for genetic counseling about these aspects and residual risks after testing puts significant strain on available genetic counseling services ^120, 121, 125^.

## Prenatal whole-exome sequencing will change our ability to identify causes of fetal birth defects

The most recent development in prenatal and reproductive testing is fetal diagnostic WES. When fetal congenital abnormalities are identified on prenatal ultrasound, karyotype and CMA reveal a diagnosis in up to 20–30% ^7, 23^, depending on the type of structural defect. For the remainder, single-gene tests or gene panels, such as testing for Noonan syndrome when there is an increased nuchal translucency in a fetus with a normal karyotype ^126, 127^, may be useful, but very recent data suggest that diagnostic WES can provide answers in a substantial proportion of the remaining cases ^7^. For WES, the majority of coding exons, which represent only 2% of the genome but contain 85% of disease-causing mutations, are sequenced. In the pediatric population, WES yields a molecular diagnosis in at least 25% of patients with a suspected genetic disorder and prior negative genetic testing ^128, 129^. Several recent case reports or small series ^128– 133^ (some embedded in larger reports) that describe diagnostic WES for fetuses or newborns with prenatally detected congenital abnormalities are now appearing ^10, 134^. Carss *et al.* report on their experience with WES on 30 prenatally or neonatally obtained samples from fetuses with congenital abnormalities but negative results on standard genetic testing. They found a genetic diagnosis in three (10%) and sequence variants of potential significance in five (17%) ^8^. More recently, Alamillo *et al.* reported relevant mutations in four of seven prenatal cases ^135^, and Drury *et al.* found a 25% total detection rate in 24 fetuses with abnormal ultrasound findings, including a definitive diagnosis in five and plausible diagnosis in one ^11^. Our early results also indicate that the detection rate of a significant genetic abnormality with prenatal exome sequencing for fetuses with single or multiple congenital anomalies is at least 30% ^18, 128^.

These combined data are very encouraging and indicate that prenatal diagnostic WES has the potential to double the number of pregnancies complicated by fetal congenital abnormalities for which a genetic etiology can be identified prenatally, but further larger studies are required.

## Concluding remarks and forecasts for the future of prenatal and reproductive genetics

The recent rapid introduction of non-invasive prenatal screening for chromosomal abnormalities has changed the practice of prenatal genetic diagnosis and screening. Although both sensitivity and specificity of cffDNA screening for fetal Down syndrome and other common aneuploidies are very high, this technique does not have the same resolution or coverage as a karyotype or CMA nor does it replace the diagnostic capability or accuracy of amniocentesis or CVS. Although laboratories have begun to add screening for other aneuploidies, such as microdeletions and duplications, there is significant concern as more rare conditions are included about adequate clinical validation, high cumulative false positive rates, resulting in unnecessary diagnostic procedures, and high false negative rates resulting in missed genetic diagnoses. Awareness of these issues by providers and patients is incomplete and marketing of cffDNA screening is highly focused on avoidance of the risk of diagnostic procedures. This may result in some patients electing for cffDNA screening when diagnostic testing is more optimal, such as in the work-up for fetal abnormalities even though prenatal CMA detects clinically significant chromosomal abnormalities in 1 to 1.7% in pregnancies without fetal anomalies and in 6% of pregnancies complicated by fetal anomalies, in addition to those chromosomal abnormalities detected by karyotyping. Thus, until non-invasive tests become more accurate and comprehensive, the growing trend of replacing diagnostic testing with cffDNA screening comes at a cost of missed prenatal genetic diagnoses. Furthermore, it is predicted that diagnostic WES to search for single-gene disorders has the potential to double the number of identified genetic causes of fetal abnormalities. Women should be counseled about the limitations of cffDNA screening in view of results from a recent meta-analysis that indicates a lower risk of diagnostic procedures than previously considered (about 1:909 for amniocentesis and 1:600 for CVS). Finally, although proof-of-principle studies have shown that it is technically feasible to non-invasively sequence the entire fetal genome, this is not currently achievable in a time- and cost-effective manner ^136, 137^. Thus, until non-invasive analysis of fetal DNA improves to the point that it will have the same accuracy as that of karyotyping and CMA on amniotic fluid or CVS samples, genetic counseling should objectively present the limitations and benefits of all currently available approaches in the context of the individual woman’s *a priori* risk, her desire for genetic knowledge about her pregnancy, and personalized risk-benefit considerations.

Since cffDNA is admixed with maternal cfDNA, it is unclear if diagnostic-level accuracy from this fetal DNA source will ever be achievable. This has sparked renewed interest by several groups in the isolation and analysis of intact fetal cells from maternal blood ^138– 145^, which contain a pure unmixed fetal genome, with a theoretical ability for similar diagnostic accuracy as that obtained through invasive diagnostic procedures. There is strong evidence that fetal cells can be recovered and analyzed, but the approach is currently labor intensive and costly and has not yet been proven to be robustly successful and adaptable to a high-throughput, relatively low-cost diagnostic testing option.

In conclusion, the advances of genomic medicine are impacting prenatal diagnosis, just like any other medical field. While these innovations offer exciting new opportunities and can empower families with increased knowledge about their reproductive risks and with decision-making autonomy, they have to be carefully introduced in an evidence-based and ethically responsible manner and monitored after implementation. Considering that many of these innovations are driven by for-profit companies, professional societies will play an increasingly important role in providing objective guidance to patients and providers.

## Abbreviations

cffDNA, cell-free fetal DNA; cfDNA, cell-free DNA; CMA, chromosomal microarray analysis; CVS, chorionic villus sampling; FISH, fluorescence *in situ* hybridization; Mb, megabase; NGS, next-generation sequencing; PPV, positive predictive value; WES, whole-exome sequencing.
